# Uveal Effusion Syndrome: Clinical Characteristics, Outcome of Surgical Treatment, and Histopathological Examination of the Sclera

**DOI:** 10.3389/fmed.2022.785444

**Published:** 2022-06-09

**Authors:** Nan Zhou, Lihong Yang, Xiaolin Xu, Wenbin Wei

**Affiliations:** Beijing Tongren Eye Center, Beijing Key Laboratory of Intraocular Tumor Diagnosis and Treatment, Medical Artificial Intelligence Research and Verification Laboratory of the Ministry of Industry and Information Technology, Beijing Tongren Hospital, Capital Medical University, Beijing, China

**Keywords:** uveal effusion syndrome, nanophthalmic eye, idiopathic ciliochoroidal detachment, clinical characteristics, outcomes of surgical treatment

## Abstract

**Purpose:**

In this study, we aimed to investigate clinical characteristics and histopathology and evaluate surgical outcomes of quadrantic lamellar-sclerectomy with sclerostomy for uveal effusion syndrome (UES).

**Design:**

Retrospective, cohort study.

**Participants:**

Overall, 106 eyes of 66 patients diagnosed with UES were treated at the Beijing Tongren Hospital between January 1, 2001 and June 26, 2021.

**Methods:**

Patients were examined by routine ophthalmologic examinations, fluorescein and indocyanine green angiography (FFA/ICGA); axial length determination; color Doppler ultrasound (CDU); ultrasound biomicroscopy (UBM), optical coherence tomography (OCT), and optical coherence tomographic angiography (SD/SS-OCTA). Quadrantic lamellar-sclerectomy with sclerostomy was performed at the equator in all patients and histopathological examination of the excised sclera was analyzed in all samples.

**Main Outcome Measures:**

The reattachment of the choroid and retina with resolution of the serous fluid, best corrected visual acuity (BCVA), choroidal thickness, and recurrence of ciliochoroidal detachment were the main outcome measures.

**Results:**

Two subgroups were identified: (1) type 1 (nanophthalmic eye), wherein the eyeball was small (average axial length 15.83 ± 1.45 mm) with high hypermetropia (average 12.6 diopters) and (2) type 2 (non-nanophthalmic eye), wherein the eyeball size was normal (average axial length 23.45 ± 1.68 mm) with or without refractive error, combined with or without systemic symptoms. Histopathologically, types 1 and 2 demonstrated similarly abnormal sclera with the disorganization of collagen fiber bundles and deposits of proteoglycans in the matrix. Quadrantic lamellar-sclerectomy with sclerostomy was effective in both types 1 and 2, inducing post-operative resolution of the subretinal fluid accumulation and increasing the useful BCVA. The choroidal thickness was significantly different before and after surgery (*P* < 0.05). Approximately 98.1% of cases attained permanent reattachment within 6 months after one operation through this procedure. The single operation success rate was 96.2%, and success with one or two operations was 100%.

**Conclusions:**

UES is caused by abnormalities of the sclera and increased resistance to transscleral fluid outflow, combined with increased choroidal thickness. Quadrantic lamellar-sclerectomy with sclerostomy is an effective treatment for UES that can rescue correct the useful visual acuity.

## Introduction

The term “uveal effusion syndrome” was first described by Schepens and Brockhurst in 1963 (1). Subsequently, in 1975, Brockhurst (2) reported that this entity condition was associated with nanophthalmos and scleral abnormality and that the congestion of the choroidal venous system caused by the compression of vortex veins by a thick sclera was the cause of subretinal fluid accumulation. He then proposed a novel surgical procedure involving vortex vein decompression (3). Based on these studies, the nanophthalmos and abnormality of the sclera were considered the pathogenic factors associated with UES.

In 1982, Gass and Jallow (4) reported idiopathic serous detachment of the retina, ciliary body, and choroid, and termed it idiopathic uveal effusion syndrome (IUES). They conjectured that this disorder was caused by scleral abnormality with enhanced resistance to the outflow of transscleral intraocular protein; they introduced (5, 6) a surgical procedure comprising scleral resection without vortex vein decompression. Trelstad et al. (7) revealed that a sclera with UES demonstrated histochemical abnormalities and their findings were supported by subsequent studies (8–12). Forrester (13) believed that IUES was a type of ocular mucopolysaccharidosis, with the initial defect resting in the proteodermatan synthesis and/or degradation by the scleral fibroblasts. Additional evidence also showed that abnormal mucopolysaccharides of the sclera played an important role in IUES pathogenesis.

These reports indicated that in UES, scleral abnormality impedes transscleral outflow of intraocular protein and fluid and compresses the vortex veins, resulting in choroidal vein congestion. Intraocular fluid accumulates in the choroidal space, leading to ciliochoroidal detachment (14–18).

However, optimum treatment methods for UES have been debated. Vortex vein decompression with scleral resection was initially advocated for the treatment of uveal effusion associated with nanophthalmos. Gass (17) believed that the treatment effect had less to do with vortex vein decompression than with scleral resection to facilitate protein and fluid drainage through the sclera. The effectiveness of scleral resection and sclerostomy were also reported in subsequent studies (19–22).

In our study, we describe the clinical characteristics of UES and classify UES eyes into two subgroups based on axial length, refractive error, and systemic symptoms. In addition, to further explore the therapeutic effectiveness for UES in Asian patients, we performed a minimum volume quadrantic surgical procedure and followed up for a long time for detecting the resolution of the ciliochoroidal detachment and the useful final vision.

## Patients and Methods

We retrospectively analyzed 106 eyes of 66 consecutive Asian patients diagnosed with primary UES treated from January 1, 2001 to June 26, 2021 at the Beijing Tongren Hospital. The study and data collection were in compliance with the principles of the Declaration of Helsinki, and written informed consent was obtained from all participants. The operative procedure was performed by senior ophthalmologists (WB. W).

Cases that met the following inclusion criteria were included: (1) the fundus showed bullous retinal detachment in the periphery without any evidence of rhegmatogenous retinal detachment; (2) retinal detachment was accompanied by annular peripheral flat or bullous ciliochoroidal detachment; (3) FA/ICGA demonstrated a leopard-spot pigmentation sign without leakage from the choroid into the subretinal space; (4) the ora serrata was easily observed without scleral depression; (5) subretinal fluid was easily shifted from its position, and (6) other causes of ciliochoroidal detachment such as prolonged hypotony, intraocular tumor, dural arteriovenous fistula, and intraocular inflammation were excluded.

The clinical data concerning patient demographics, associated ocular and systemic disease, treatment history, ocular symptoms, BCVA, and intraocular pressure (IOP), axial length (by A-scan or IOL-master), standard ocular CDU (since 2010), UBM (since 2013), FFA/ICGA, choroidal thickness (by EDI-OCT/OCTA [μm], since 2010), as well as OCT and SD/SS-OCTA (since 2010) were collected. The surgical findings including intraoperative and post-operative period were noted. A record of the histopathological features was listed. Long-term outcomes, in terms of reattachment of the choroid and retina, BCVA, choroidal thickness, surgical side effects, and recurrence of UES, were assessed.

### Operative Procedure

Surgical treatment was performed in all eyes included in this study, the main surgical procedure was as Gass described previously (5), but we modified and adopted minimum volume standards for surgical procedures. We performed lamellar-sclerectomy with sclerostomy at two sites in the temporoinferior and nasoinferior quadrants during the initial surgery; if recurrence occurred, we performed additional surgeries at the equator in the supratemporal and supranasal quadrants following a similar procedure.

**Figure 1:**
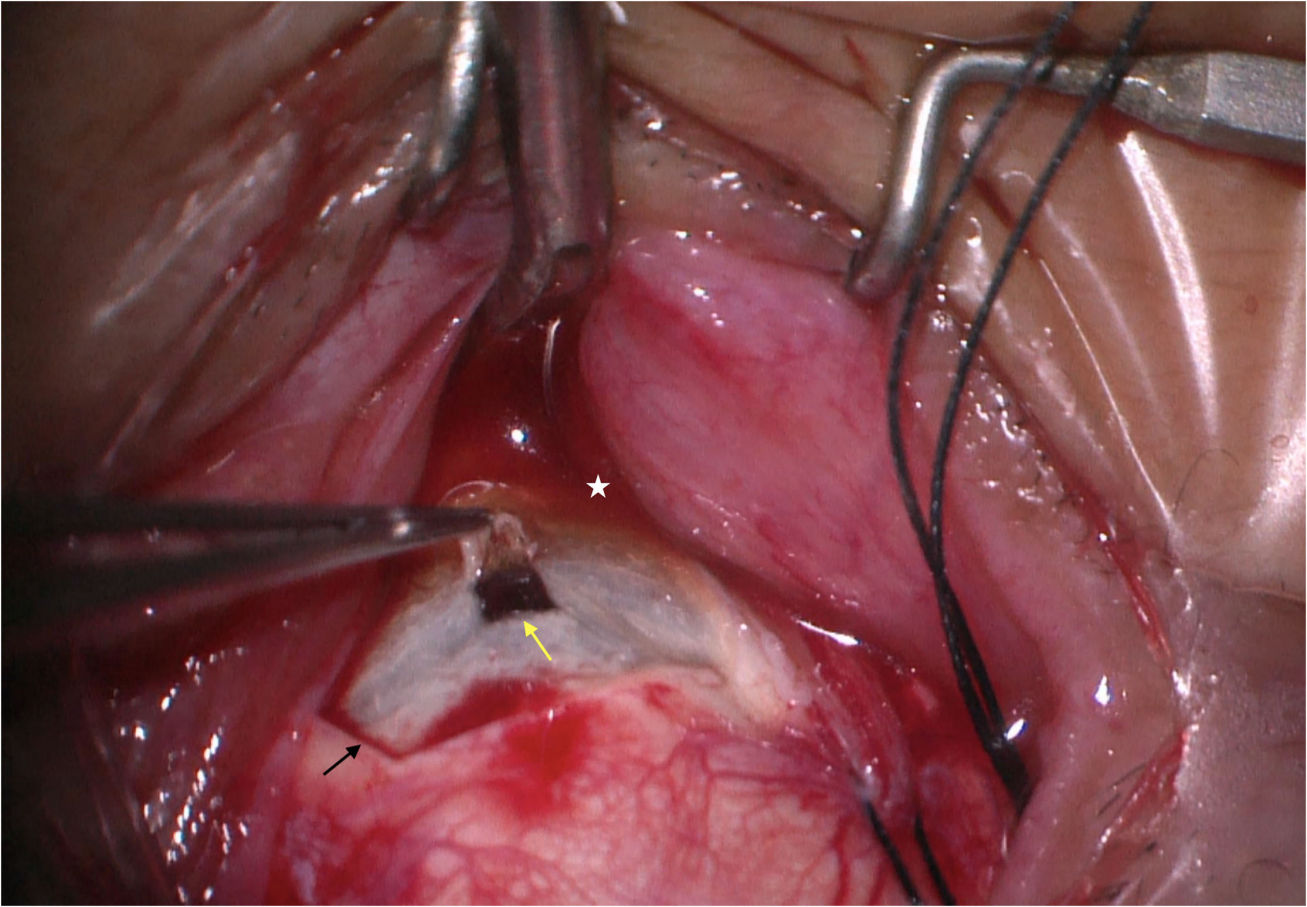
Quadrantic lamellar-sclerectomy with sclerostomy. At the equator of each quadrant, we created a two-thirds thickness scleral incision measuring 6 mm × 4 mm (black arrow), on the center of each sclerectomy bed, the sclerostomy was excised in pieces measuring 2 mm × 2 mm (yellow arrow), and the choroid was exposed. The image was taken immediately after the sclerostomy was completed. Note that the choroid is exposed and abundant yellow serous suprachoroidal fluid was spontaneously released (white star).

At the equator of these two quadrants, extending from immediately behind the extraocular muscle insertions to the vortex veins, we created a two-third thickness of scleral incision measuring ~5–6 mm × 3–4 mm (Figure 1) and performed sclerectomies in each quadrant. On the center of each sclerectomy bed, the sclerostomy that was excised in pieces measuring ~2 mm × 2 mm and the choroid were exposed. When sclerectomy was performed and the choroid was exposed, abundant serous suprachoroidal fluid was spontaneously released. If IOP was decreased during choroidal drainage, a small amount of balanced salt solution (BSS) (0.5–1.5 ml) was injected with a 30-gauge needle at a site 3.0 to 3.5 mm from the limbus into the eyeball to maintain the IOP. Tenon's capsule and the bulbar conjunctiva were closely sutured using 8-0 Vicryl sutures. We did not perform the vortex vein decompression. All excised scleral pieces were analyzed via histopathological examinations.

Patients were followed up regularly by previously described ophthalmologic examinations on months 1, 2, and 3 after surgery, and then every 6 months thereafter during the follow-up visits. In addition, FA/ICGA and EDI-OCT/OCTA were repeatedly examined.

### Statistical Analysis

Data collected on continuous scale, including age (years), axial length (millimeters) and choroidal thickness (μm), were expressed as mean, median, minimum, and maximum. Student's *t*-test was performed to compare the choroidal thickness and BCVA measurements pre- and post-operation. *P*-value <0.05 was considered to be significantly different. All analyses were performed using Stata version 15.0 (StataCorp. LLC, College Station, TX, USA).

## Results

In all, 106 eyes of 66 patients (Asian/Chinese) with UES were included in this study and underwent quadrantic lamellar-sclerectomy with sclerostomy. The mean patient age was 36.08 ± 12.67 years (median, 43; range, 26–62). To evaluate the effect of surgical management on the affected eyes, we divided patients with UES into two subgroups according to the following criteria: type 1, nanophthalmic eye, axial length <19.0 mm, with high grade of hypermetropia in refraction; type 2, non-nanophthalmic eye with abnormal sclera, normal axial length (≥21.0 mm), with or without accompanied systemic symptoms. During the time of surgery, types 1 and 2 demonstrated rigid and thickened sclera. CDU showed the highest scleral thickness of 2.1–3.2 mm.

Among the 106 eyes in 66 patients with UES, type 1 was found in 62 eyes (31 patients) and type 2 in 44 eyes (35 patients). Among them, 30 eyes (29%) were previously treated with periocular corticosteroids (triamcinolone acetonide or methylprednisolone) and showed no effective response. The median follow-up duration was 65.3 months (range, 12–122 months). Clinical data of the patients and eyes are listed in Table 1.

**Table 1:** Descriptive characteristics of patients with UES of type 1 and type 2.

**Characteristics**	**Type 1 (nanophthalmic eye) *n*1 = 31 patients, *n*2 = 62 eyes**	**Type 2 (non-nanophthalmic eye) *n*1 =35; *n*2 = 44 eyes**
Age (yes)		
Mean	35.86 ± 12.22	44.68 ± 8.18
Median (range)	31 (26–62)	43 (32–62)
Sex, No. (%)		
Female (%)	42 (13/31)	57 (20/35)
Male (%)	58 (18/31)	43 (15/35)
Laterality	100%	26 (9/35)
Right eye (%)	/	40 (21/52)
Left eye (%)	/	60 (31/52)
Axial length (mm)		
Mean	15.83 ± 1.45	23.45 ± 1.68
Median (range)	15.16 (13.85–18.03)	24.03 (21.4–26.20)
Average refractive error (D)	+12.6	<1.00
Closed angle glaucoma (%)	97 (60/62)	None
Systemic symptoms (*n*, %)	None	polyarteritis nodosa (1, 2.8%), psoriasis (2, 5.7%), demyelinating disease (1, 2.8%), rheumatoid arthritis (2, 5.7%), Sjogren Syndrome (1, 2.8%), chronic dermatitis (1, 2.8%), vasculitis (2, 5.7%), facial nevus flammeus (port-wine stain) (1, 2.8%)
Episcleral vessel dilation (%)	None	81.8% (36/44)
Recurrences during follow-up (%)	6 (4/62)	None

*UES, uveal effusion syndrome; yes, years; D, diopter*.

### Clinical Characteristics of UES Patients

#### Type 1 (Nanophthalmic Eye)

**Figure 2:**
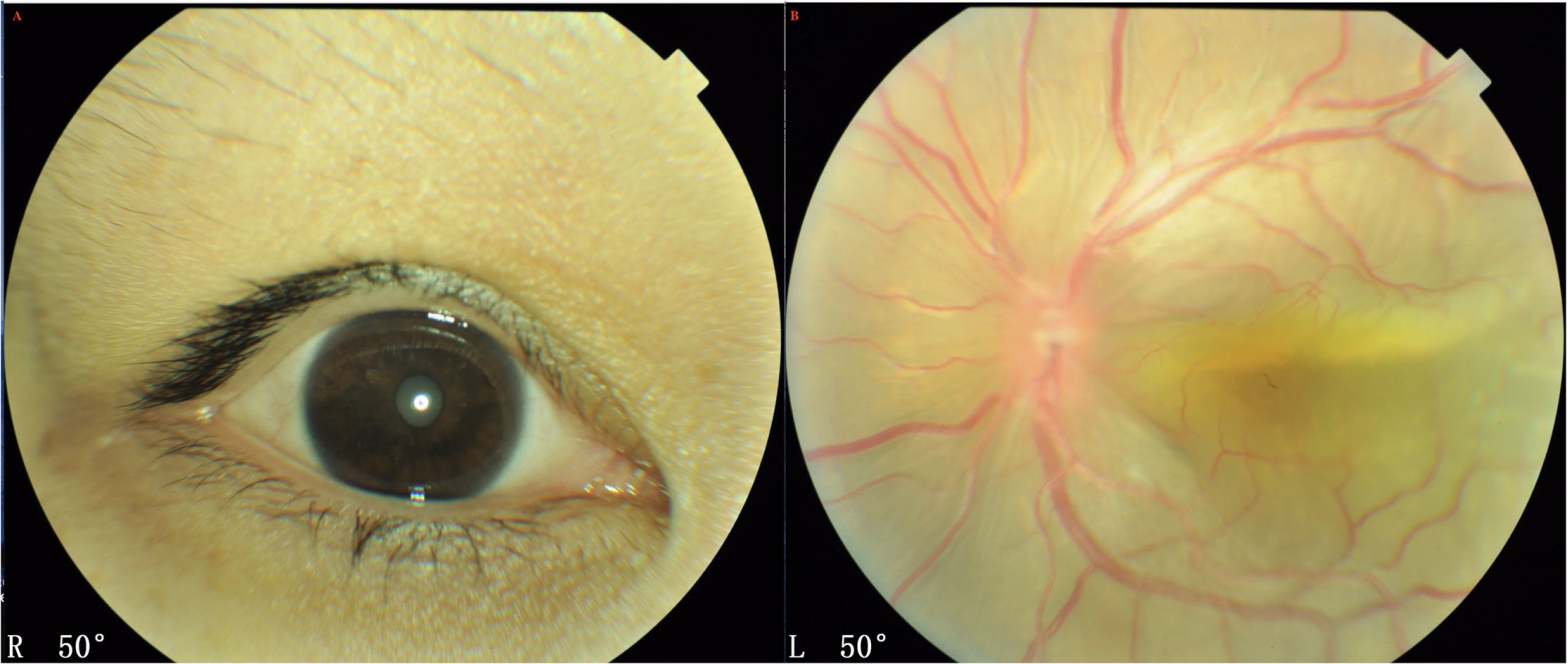
A 27-year- women with UES type 1. **(A)** Patients presented with nanophthalmos eyes. **(B)** Exudative retinal detachment accompanied by annular peripheral ciliochoroidal detachment was observed (Imagenet 6, Topcon, Japan).

There were 62 eyes in 31 patients in this group and all patients presented with bilateral nanophthalmos eyes (Figure 2A). The mean age was 35.86 ± 12.22 years (median, 31; range, 26–62 years), with 18 males and 13 females (Table 1). All patients were in good health systemically. Axial length was 15.83 ± 1.45 mm (median, 15.16, ranged, 13.85–18.03 mm), and the axial lengths showed <1 mm difference between both eyes of a single patient. Both eyes demonstrated high hypermetropia, with an average of +12.6 D (ranging, +8.9 to +18.0 D).

Further, 60 eyes (97%) of 30 patients showed a peripheral shallow anterior chamber because of a swollen iris, and underwent YAG laser iridectomy, and IOP was within normal limits in all eyes post-operatively. Exudative bullous retinal detachment accompanied by annular peripheral ciliochoroidal detachment was observed in the unilateral eye (Figure 2B); and slight annular ciliochoroidal detachment and choroidal folds that were not significant were seen in the contralateral eye.

**Figure 3:**
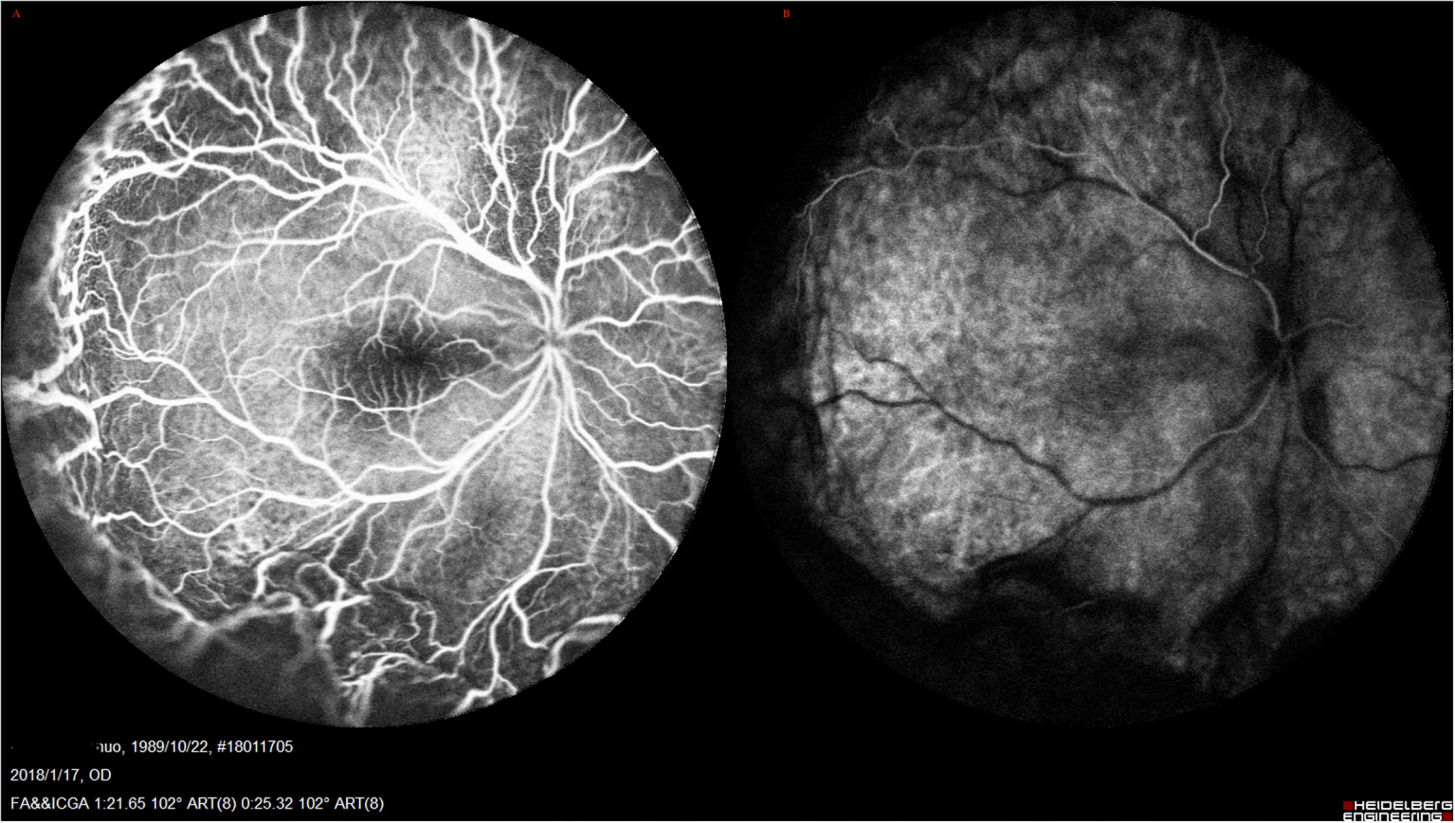
UES in Type 1. **(A)** FFA (Spectralis; Heidelberg Engineering, Inc.) revealed the retinal vein dilation with leopard-spot sign of granular hyperfluorescence of the retinal pigment epithelium (RPE) was observed in the posterior pole and inferior quadrants. **(B)** ICGA revealed diffusely granular marked choroidal hyperfluorescence at the early stage, which increased with time and persisted until the late stage as diffuse intense choroidal hyperfluorescence.

Both eyes demonstrated a small optic disc with slight papilledema, which was commonly observed in the highly hypermetropic eye. FFA did not reveal any abnormality except retinal vein dilation in 33 eyes; however, a leopard-spot sign of granular hyperfluorescence of the retinal pigment epithelium (RPE) was observed in the posterior pole and inferior quadrants in all eyes (Figure 3A). ICGA revealed diffusely granular marked choroidal hyperfluorescence at an early stage, which increased with time and persisted until the late stage as diffuse intense choroidal hyperfluorescence (Figure 3B). The abnormal ICGA findings in the affected eye were the same as those observed in the fellow eye. UBM showed the presence of ciliary body edema and detachment in all eyes, which led to an anterior rotation of the ciliary body. CDU clearly demonstrated small eye sizes and remarkably thickened sclera (mean 2.5 mm), with the accumulation of subretinal fluid.

#### Type 2 (Non-nanophthalmic Eye)

There were 44 eyes of 35 patients in this group; most of the patients (26/35, 74.3%) showed unilateral UES. The mean age was 44.68 ± 8.18 years (median, 43; range, 32–62 years), with 15 males and 20 females (Table 1). Axial length was 23.45 ± 1.68 mm (median, 24.03, range, 21.4–26.20 mm). Both eyes of a single patient revealed almost the same normal axial lengths. The difference in the axial length in both eyes was within 1.0 mm. Refractive errors of these eyes were <1.00 D, without any high hyperopia.

**Figure 4:**
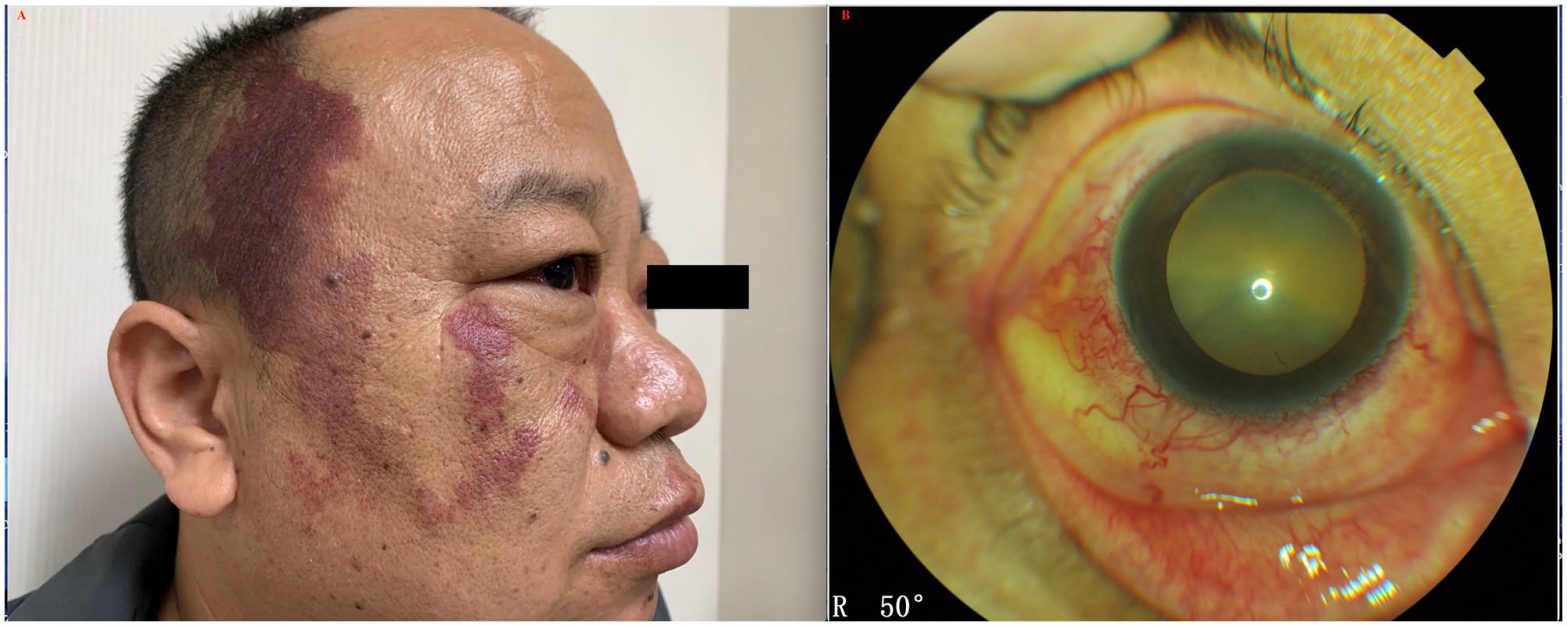
**(A)** Patient with UES type 2 and facial nevus flammeus (port-wine stain). **(B)** External eye examination revealed episcleral vessel dilation; anterior chamber was free of cells.

**Figure 5:**
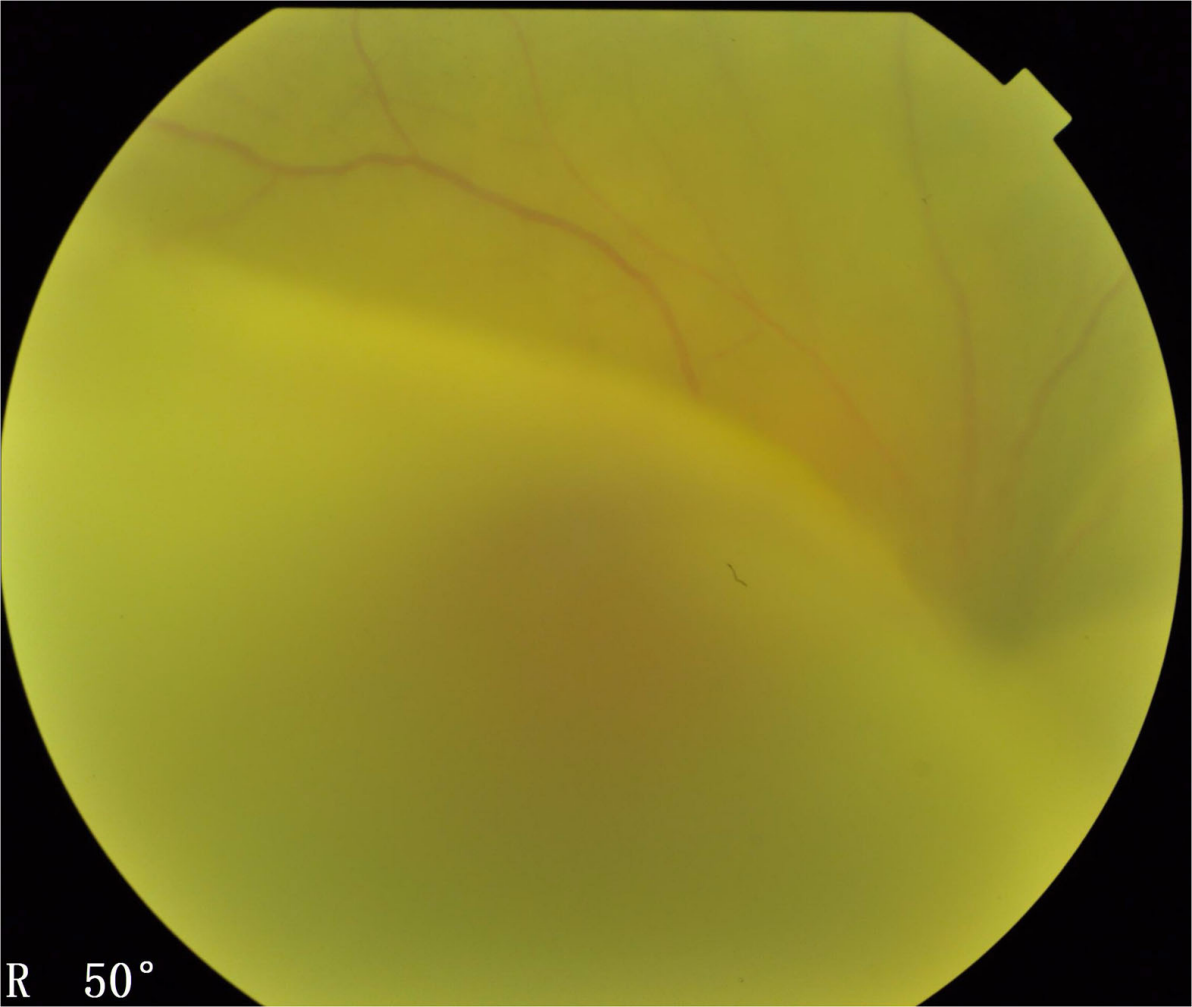
Type 2 UES. The fundus examination (Imagenet 6, Topcon, Japan) demonstrated typical manifestations of exudative bullous retinal detachment associated with ciliochoroidal detachment similar to those observed in type 1, except that there was no papilledema.

**Figure 6:**
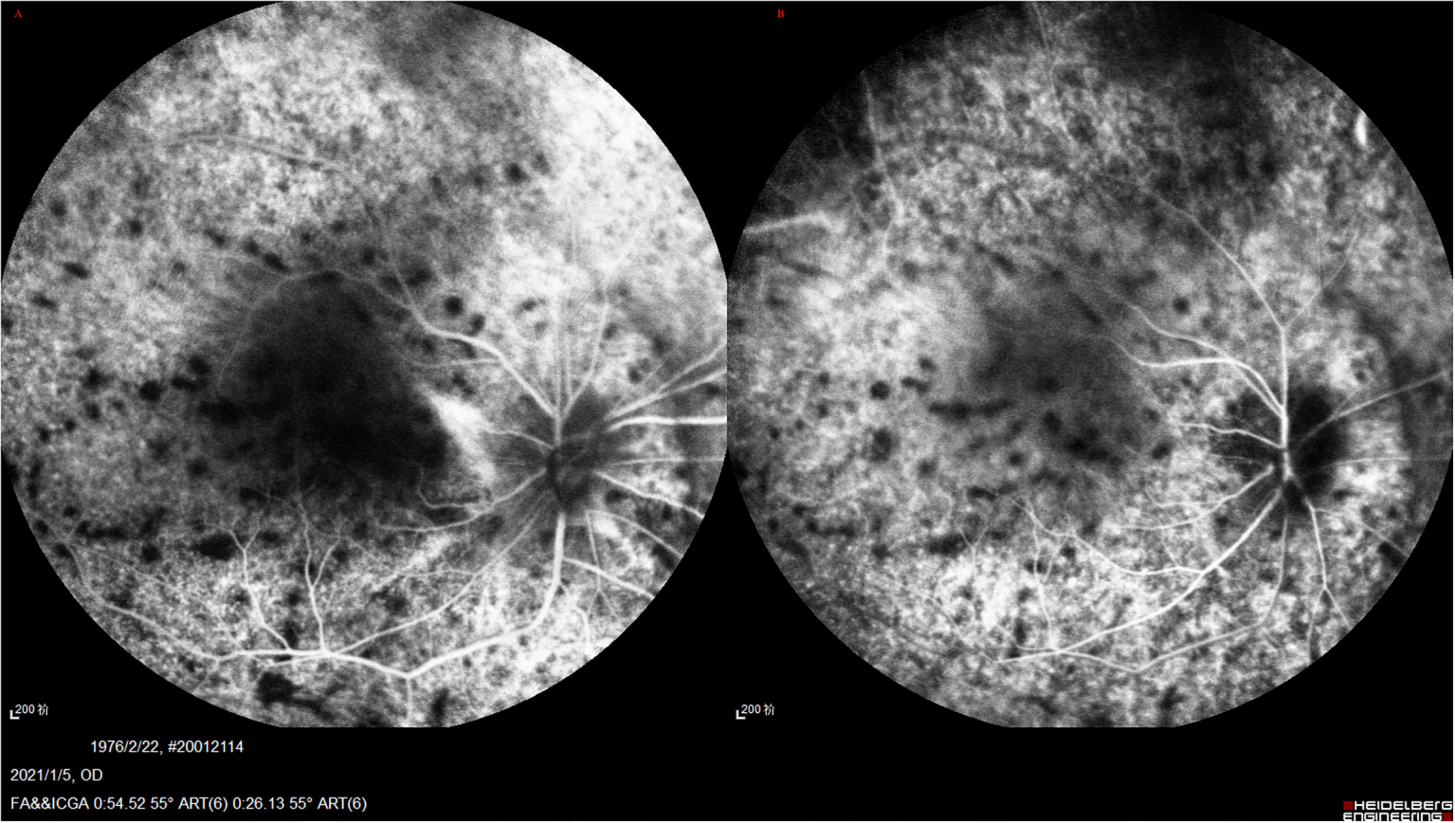
**(A,B)** UES in type 2. FA/ICGA (Spectralis; Heidelberg Engineering, Inc) findings showed diffused patchy hyperfuluorescence and leopard-spot sign, which were almost similarly observed in type 1, without dye leakage in any eye.

**Figure 7:**
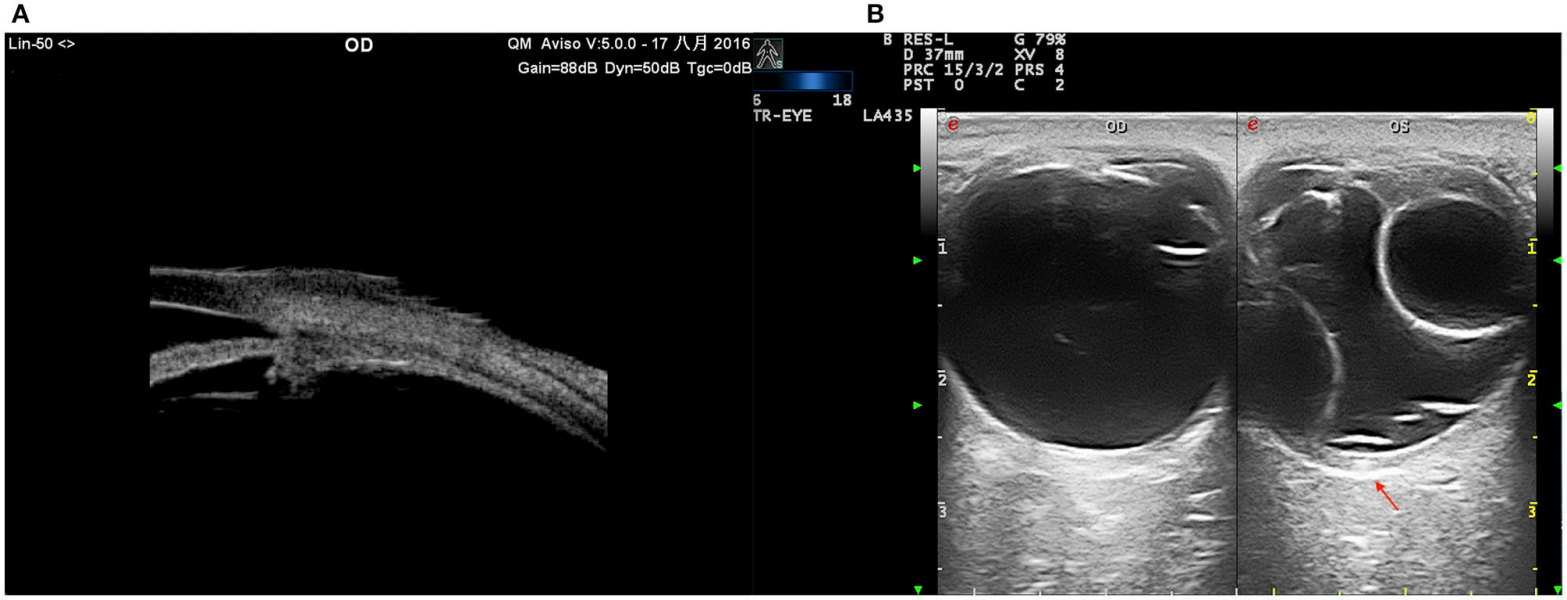
UES in type 2. **(A)** UBM examination showing effusion of the ciliary body and thickened sclera. **(B)** CDU (TomTec Imaging System, Germany): annular bullous choroidal detachment, remarkably thickened sclera (red arrow).

The accompanying systemic symptoms were polyarteritis nodosa in 1 patient, psoriasis in 2 patients, demyelinating disease in 1 patient, rheumatoid arthritis in 2 patients, Sjogren Syndrome in 1 patient, chronic dermatitis in 1 patient, vasculitis in 2 patients, and facial nevus flammeus (port-wine stain) in 1 patient (Figure 4A). External eye examination revealed episcleral vessel dilation (36/44, 81.8%) (Figure 4B); anterior chamber was free of cells; IOP was normal in all eyes. Fundus examination demonstrated typical manifestations of exudative retinal detachment associated with ciliochoroidal detachment similar to those observed in type 1, except that there was no papilledema (Figure 5). FA/ICGA findings showed diffuse patchy hyperfluorescence and leopard-spot signs, which were almost similar to those observed in type 1, without dye leakage in any eye (Figures 6A,B). UBM showed the presence of a ciliochoroidal effusion and ciliary body detachment, similar to type 1, in the affected eyes. In all patients, CDU showed normal-sized eyes, with marked thickening of the sclera (mean 2.7 mm) and choroidal and retinal detachment in all eyes (Figure 7).

## OCT and OCTA

The choroidal thickness and macular areas in both eyes of UES patients were evaluated via OCT and OCTA. All eyes (type 1 and type 2) showed pachychoroid.

**Figure 8:**
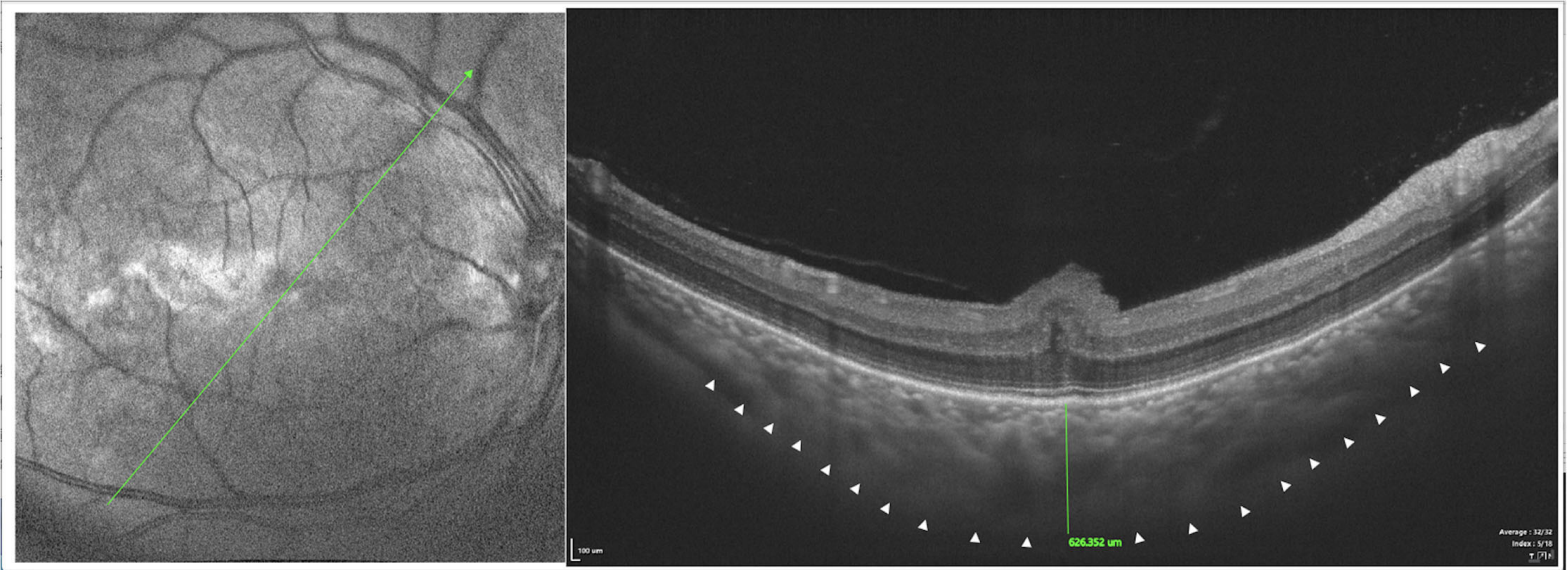
SS-OCT (SS-OCT, VG200D, SVision Imaging, Ltd., China) in type 1 showed that each retinal layer was uneven and wavy with pachychoroid, leopard-sign spots in the fundus corresponding to accumulation of outer retinal material, which presented as focal thickening of the RPE layer.

**Figure 9:**
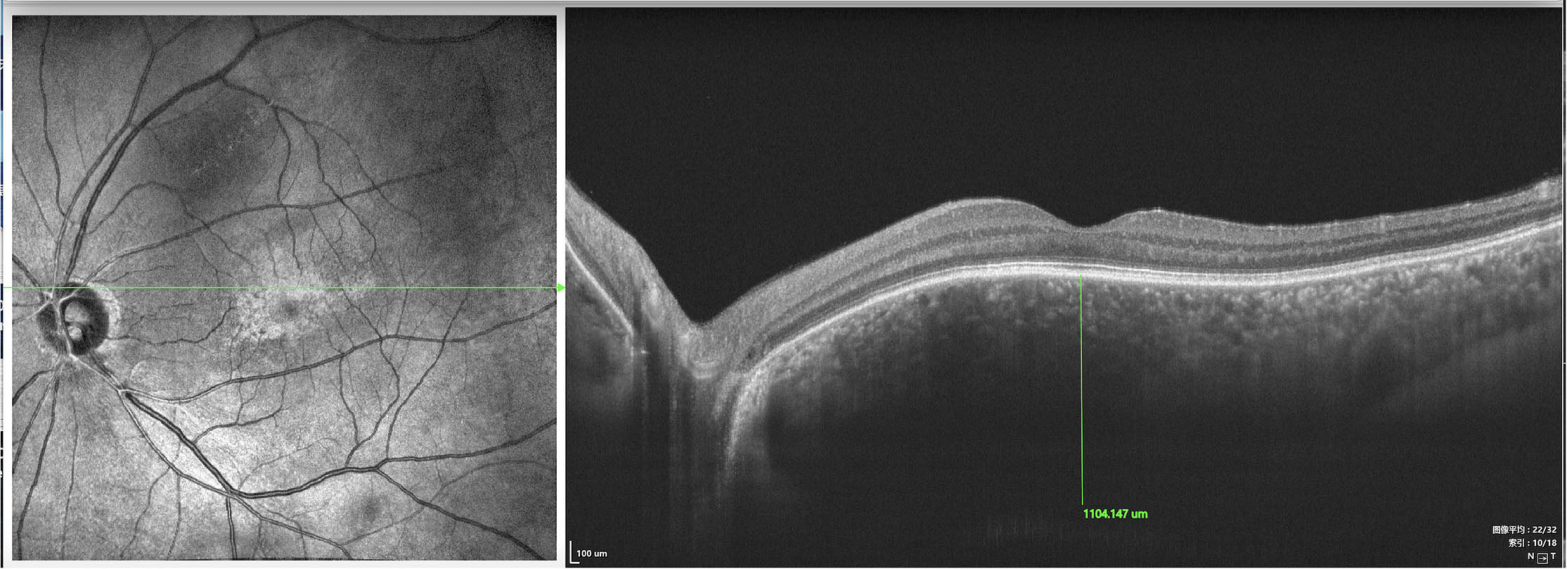
SS-OCT (SS-OCT, VG200D, SVision Imaging, Ltd., China) in type 2 showed that each retinal layer was even with the pachychoroid, leopard-sign spots in the fundus corresponding to accumulation of outer retinal material, which presented as focal thickening of the RPE layer.

In type 1, EDI-OCT was performed in 42 eyes of 21 patients and a choroidal thickness of 869.46 ± 8.33 μm (range, 381.20–878.58 μm) was observed (Figure 8); both eyes of the same patient showed almost the same choroidal thickness (Figure 7B); in type 2, EDI-OCT was performed in 38 eyes of 19 patients; a choroidal thickness of 816.56 ± 10.21 μm (range, 589.63–1075.61 μm) was observed in the affected eye and 781.23 ± 9.33 μm (range, 692.42–1104.15 μm) in the fellow eye (Figure 9). Both eyes of the same patient showed almost the same choroidal thickness.

SD-OCT showed that each retinal layer was uneven and wavy; leopard spots were observed in the fundus corresponding to the accumulation of outer retinal material, which presented as focal thickening of the RPE layer. B-scan of SS-OCT showed the scleral thickness of ~3 mm (Figure 8), consistent with the CDU measurements.

**Figure 10:**
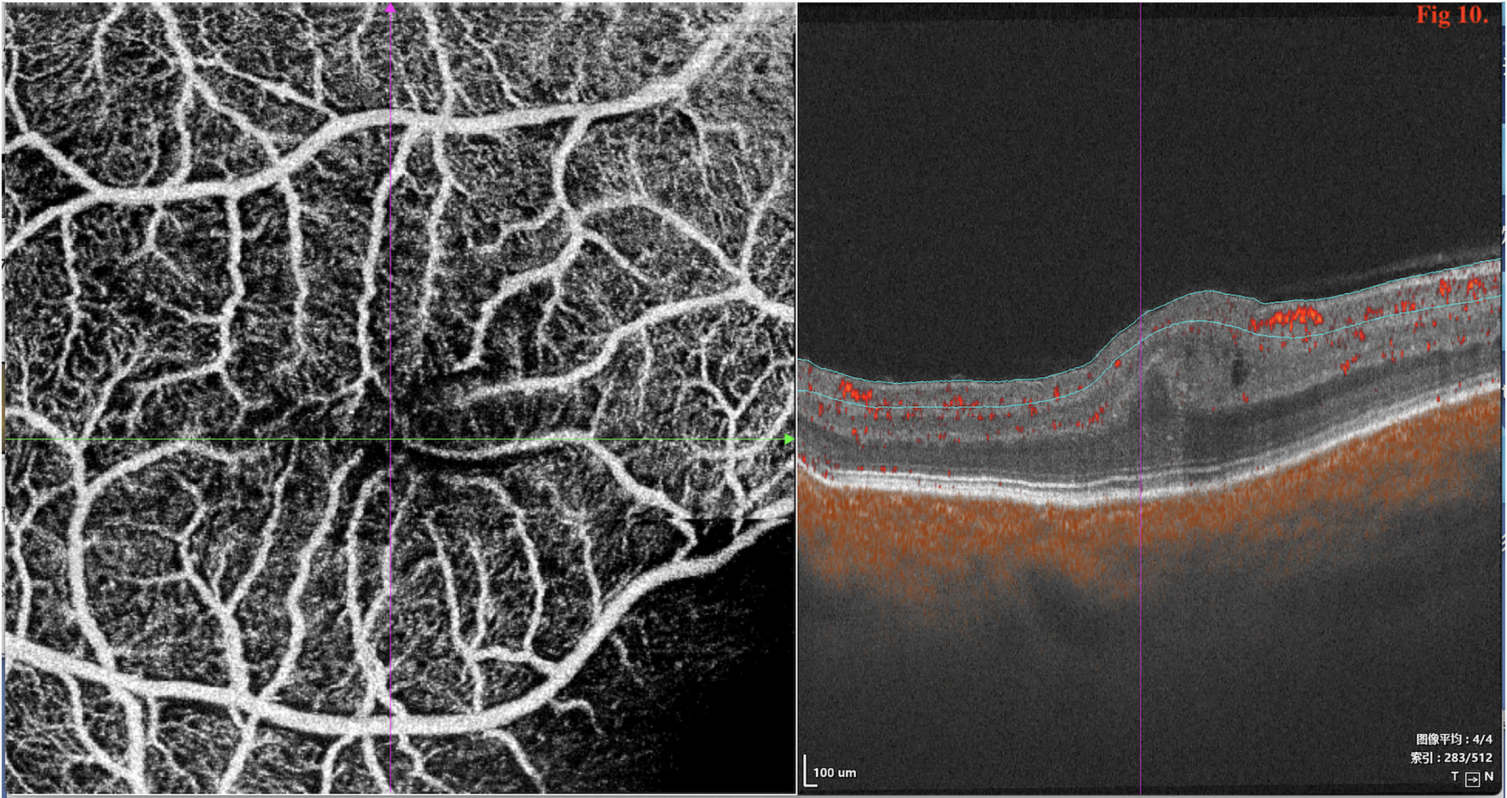
SD/SS-OCTA (SS-OCT, VG200D, SVision Imaging, Ltd., China) in type 1 revealed that the macular morphology was crowded and showed macular dysplasia with decreased size of foveal avascular zone (FAZ).

SD/SS-OCTA was performed in 18 type 1 eyes and revealed capillary tortuosity, thickened subfoveal choroid, crowded macular morphology, and macular dysplasia with attenuation of foveal avascular zone (FAZ) (Figure 10). The restoration of photoreceptor and RPE damage, flattening of crowded macular morphology, and expansion of attenuation of FAZ were observed by SS-OCT/OCTA with time during follow-up post-operatively (Supplementary Figures 1, 2).

**Figure 11:**
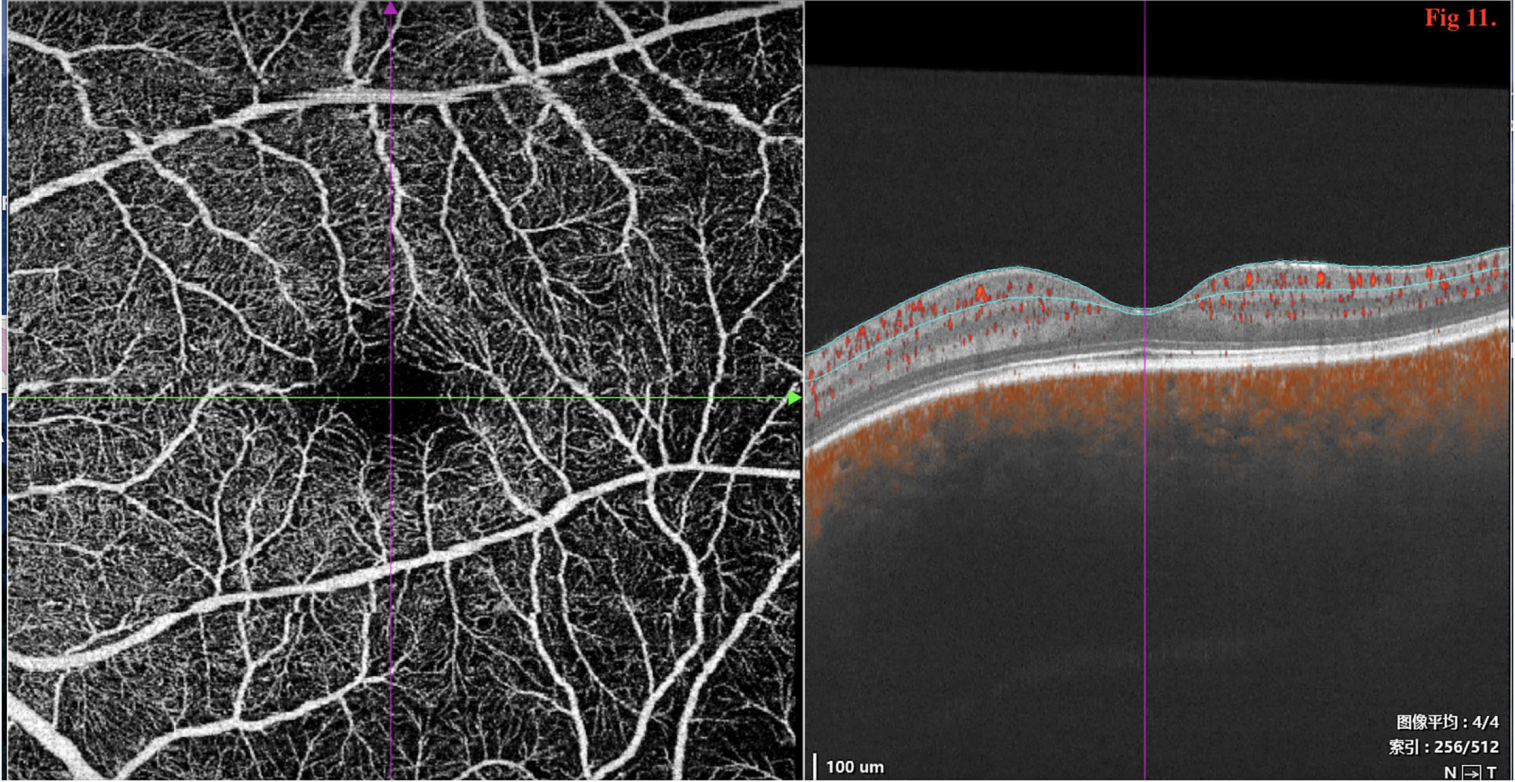
SD/SS-OCTA (SS-OCT, VG200D, SVision Imaging, Ltd., China) revealed that the macular morphology was normal with no decrease in the size of foveal avascular zone (FAZ).

SD/SS-OCTA was performed in 16 eyes in type 2 and revealed the normal FAZ size and retinal layers (Figure 11); however, pachychoroid was seen in both eyes.

## Surgical Management and Outcome

We performed quadrantic lamellar-sclerectomy with sclerostomy at two sites at the equator in the inferior quadrants in all eyes and the procedure was as described above (Figure 1). There were no surgical side effects, such as hemorrhage, infection, iatrogenic retinal tear, and aggravation of cataract, during the intraoperative or post-operative period. Of the eyes tha underwent one scleral thinning surgery, complete resolution of subretinal and supraciliochoroidal fluid was observed within 6 months in 98.1% of eyes, and the mean time of retinal flattening was 1.2 months (range: 0.5–2.5 months).

### Type 1

We performed surgery in 62 eyes. During surgery, we noted that the eyeballs were small sized, with insertions of the rectus muscles and the equator located unusually anteriorly; the equator located 8 to 10 mm posterior to the limbus. The sclera were abnormally rigid and thick. Thickness of the sclera at the equator was more than 2.0 mm, which was consistent with the CDU results.

**Figure 12:**
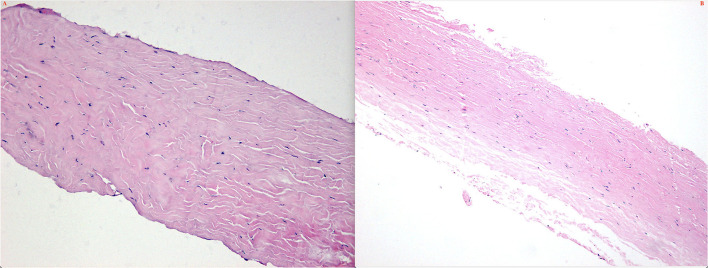
Histochemical features of the scleras in type 1 **(A)** and type 2 **(B)**. Note the collagen fibers bundles was variable diameters, arranged irregularly; spaces between collagen fibers bundles were enlarged.

After surgery, ciliochoroidal and retinal detachment resolved with complete resolution of subretinal fluid within 1.0 to 2.5 months in all nanophthalmic eyes (Figure 12).

Among patients who underwent bilateral surgery, the mean interval between surgeries for the two eyes was 9.85 ± 11.50 months (median 8, range 1–36 months).

Two eyes of 2 patients showed recurrence of UES 2 years later and 2 eyes of 2 patients showed recurrence 3 years later; we performed the same surgery at two sites at the equator in the upper quadrants. After reoperation, the choroidal and retinal detachment were completely resolved without any signs of recurrence at the last follow-up. In addition, 3 eyes (5%) of 2 patients in type 1 group showed reattachment spontaneously without surgery (follow-up duration, 11 months to 8 years).

One year after surgery, FA/ICGA was performed in all patients in whom the retina was completely reattached. Angiography demonstrated marked hyperfluorescence in the choroidal background fluorescence during the early to late phase in both the affected and unaffected eyes. These findings were similar to those observed before the surgery.

In the type 1 group, BCVA at presentation was 20/100 to 20/200 in 20 (32.2%) eyes, and ≤ 20/200 in 42 (67.7%) eyes; final BCVA was 20/80 in 22 (35.5%) eyes, 20/100 to 20/200 in 33 (53.2%) eyes (Table 2).

**Table 2:** Change in best corrected visual acuity (BCVA) of patients with UES before- and post-surgery to the last follow-up visit available.

**BCVA-before surgery (%)**	**Type 1 (nanophthalmic eye) *n*1 = 31 patients, *n*2 = 62 eyes**	**Type 2 (non-nanophthalmic eye) *n*1 = 35; *n*2 = 44 eyes**
<20/200	42 (67.7%)	36 (86.4%)
20/100–20/200	20 (32.2%)	8 (18.1%)
BCVA-post surgery		
<20/200	7 (11.3%)	4 (9.0%)
20/200–20/133	33 (53.2%)	35 (79.5%)
20/80	22 (35.5%)	5 (11.4%)

*BCVA, best corrected visual acuity*.

### Type 2

In 44 eyes, we performed quadrantic lamellar-sclerectomy with sclerostomy at two sites at the equator in the lower quadrants. During surgery, the eyeball was normal in size; however, the sclera was abnormally rigid and thickened in all eyes. The sclera at the equator was more than 2.5 mm thick.

**Figure 13:**
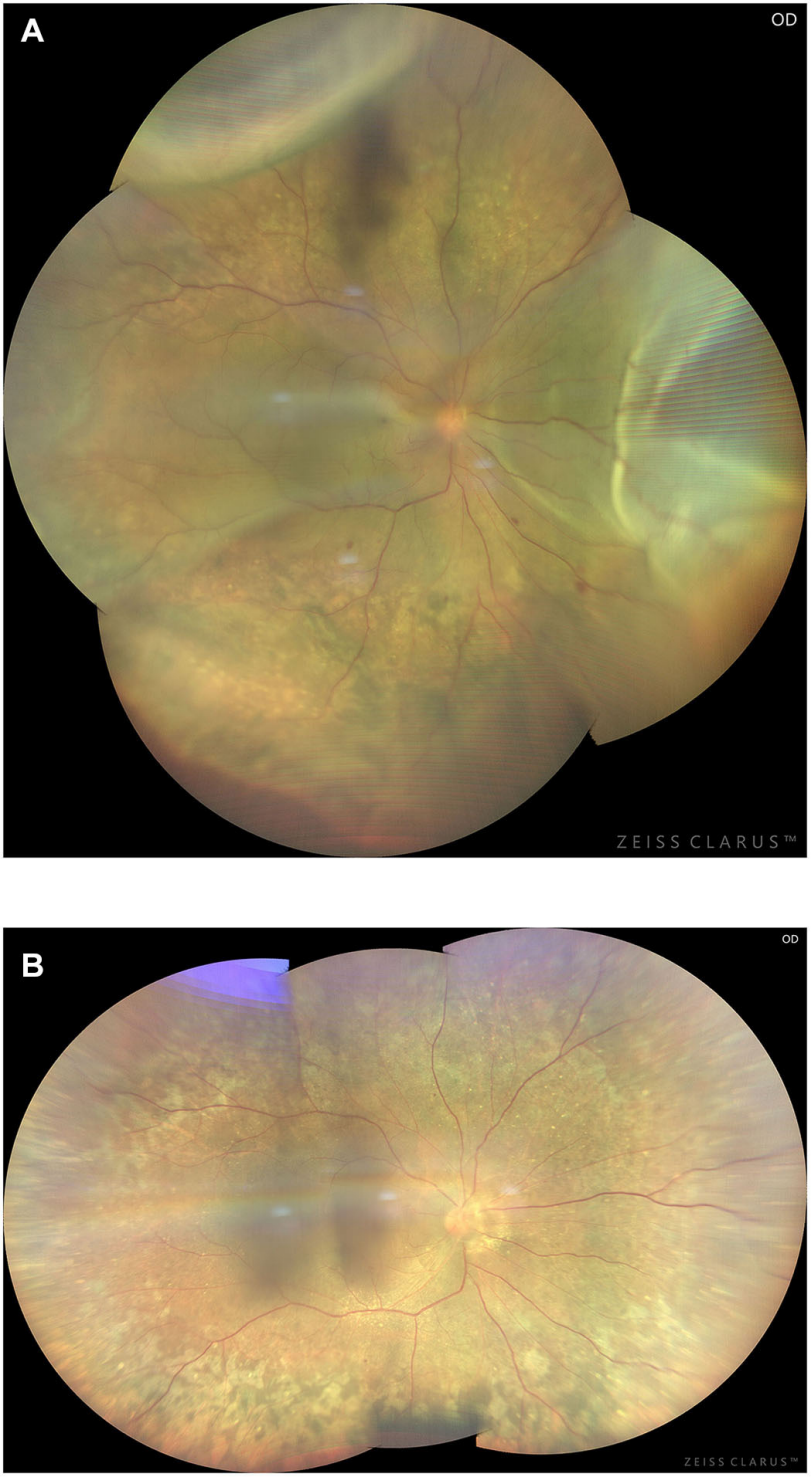
Preoperative **(A)** and 3-month postoperative **(B)** retinal images (montage, CLARUS 500; Carol Zeiss) of an eye with type 2 UES. Note retinal detachment involving the macula and choroidal swelling extending posterior to the equator in the superior quadrants **(A)**. Total resolution of the retinal detachment and choroidal swelling **(B)** was achieved 3 months after quadrantic lamellar-sclerectomy with sclerostomy in the inferior quadrants.

All procedures were successful and no recurrence of UES was observed at the last follow-up. In 2 patients, the unaffected eyes showed retinal and choroidal detachment caused by UES, 9 and 11 months after the affected eye was operated, respectively. The surgery was performed and was successful in these two patients (Figure 13).

In the type 2 group, BCVA at presentation was 20/100 to 20/200 in 8 (18.1%) eyes, and ≤ 20/200 in 36 (86.4%) eyes; final BCVA was 20/80 in 5 (11.4%) of eyes, 20/100 to 20/200 in 35 (79.5%) (Table 2).

In type 2, we disclosed that 26 patients had a monocular disease and 9 had a bilateral disease. A total of 9 patients who had monocular involvement at initial examination developed disease of the contralateral eye at a mean interval of 9.0 months (range, 8–12 months).

There was no difference in choroidal thickness and BCVA between the two groups (*P* > 0.05) post-operatively. The final BCVA was not related to the time of retinal choroidal detachment, but related to the early subretinal fluid removal and photoreceptor damage.

At the 6-month follow-up, the choroidal thickness in both groups after operation was lower than that before operation (*P* < 0.05). At the last follow-up, the mean choroidal thickness in the type 1 group before and after operation was 869.46 ± 8.33 μm and 599.53 ± 9.16 μm, respectively; in the type 2 group, the mean choroidal thickness of the affected eyes before and after operation was 836.56 ± 10.21 μm and 713.32±8.43 μm, respectively (Table 3). There was significant difference in the before and after operation values between these two groups (*P* < 0.05). After age and gender matching, there were no significant differences in the choroidal thickness of affected eyes between type 1 and type 2 UES patients (*P* > 0.05).

**Table 3:** Change in choroidal thickness of patients with UES before- and post-surgery to the last follow-up visit available.

	**Type 1 (nanophthalmic eye) *n*1 = 21 patients, *n*2 = 42 eyes**	**Type 2(non-nanophthalmic eye)**	
		***n*****1** **=** **19;** ***n*****2** **=** **38 eyes**	
Choroidal thickness (um)	Affected eyes	Affected eyes	Fellow eyes
Before-surgery	869.46 ± 8.33	816.56 ± 10.21	781.23 ± 9.33
Post-surgery	599.53 ± 9.16	713.32 ± 8.43	/

## Histopathological Examination of the Sclera

**Figure 14:**
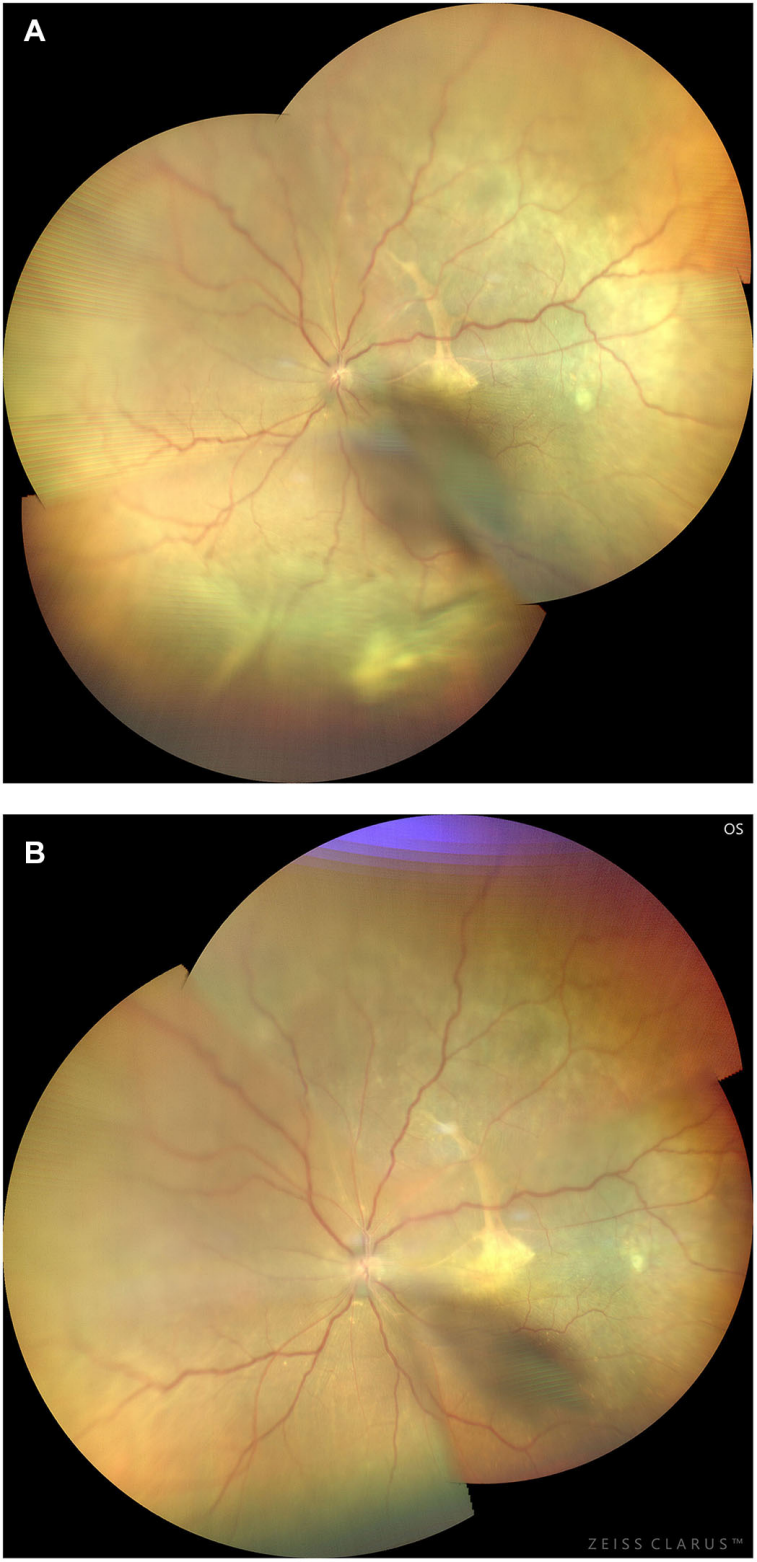
Preoperative **(A)** and 3-month postoperative **(B)** retinal images (montage, CLARUS 500; Carol Zeiss) of an eye with type 1 UES. Note that the retinal detachment with choroidal swelling extending posterior to the equator in the inferior quadrants, and a subretinal proliferative membrane and macular fold are observed **(A)**. Total resolution of the retinal detachment and choroidal swelling **(B)** was achieved 3 months after quadrantic lamellar-sclerectomy with sclerostomy in the inferior quadrants.

Surgically excised scleral pieces of type 1 and type 2 were examined histopathologically. Pathologically, collagen fiber bundles in the thickened sclera demonstrated markedly irregular arrangement and the widths of the bundles varied (Figures 14A,B). Deposits of matrix between the bundles were remarkable in all eyes.

Histochemical features of the sclera showed that collagen fiber bundles were of variable diameters and arranged irregularly and spaces between collagen fibers bundles were enlarged; however, the Alcian blue staining (glycosaminoglycans) of the matrix in type 1 and type 2 eyes was negative.

## Discussion

To the best of our knowledge, our study, 106 eyes of 66 patients with UES, reports the largest case series concerning UES including nanophthalmos and non-nanophthalmos (idiopathic). After undergoing one scleral thinning surgery, complete resolution of subretinal and supraciliochoroidal fluid was observed within a mean time of 1.2 months in 98.1% of eyes. During a mean follow-up duration of 65.3 months, the final BCVA improved or stabilized in 89.6% of eyes; however, OCT/OCTA imaging revealed that the extent of improvement was limited by photoreceptor and pigment epithelial damage resulting from macular dysplasia or chronic retinal detachment. The OCTA role in defining macular status and visual potential is consistent with Mansour's (23) findings in nanophthalmos. In addition, in our nanophthalmos group, secondary glaucoma combined with optic atrophy was also the main reason for poor final vision.

In the present study, we divided the eyes of patients with primary UES into two groups mainly according to the axial length: type 1 and type 2 UES. Type 1 and 2 UES showed incompletely same clinical characteristics but similar histologic appearance, except for the axial length and refractive error (Table 1). These results indicate that both types are subtypes in the same category.

In incomplete conformity with previous studies (1, 2, 4), several special clinical features were noted in a large proportion of our Asian patients: type 1 showed bilateral involvement, protracted course, elevated IOP accompany closer of anterior chamber, and absence of known systemic disorders; type 2: male sex and unilateral involvement, middle age at onset, normal IOP, dilated episcleral veins, and no vitreous cells, but may be accompanied with different systemic symptoms. Ancillary testing typically revealed characteristic FFA abnormalities consistent with leopard-spot RPE alterations. CDU showed evidence of diffuse choroidal thickening, and similarly, OCT/OCTA showed pachychoroid in type 1 and type 2 UES. The choroidal thickness significantly changed before and after surgery.

Subsequent single recurrences in 0.4% of eyes in type 1 over a mean follow-up duration of 65.3 months responded to reoperation. No recurrence was observed in type 2. Only 3 eyes of 2 patients (0.3%) in type 1 occurred reattachment spontaneously without surgery (range of follow-up, 3 months to 2 years). However, in the absence of an unoperated control group with similar disease severity, we cannot entirely confirm whether the reattachments observed in our cases were spontaneous and unrelated to the surgical procedure; alternatively, this may be a phase in the natural history of the disorder.

The pathogenesis of UES is unclear, possibly related to congenital anomaly of the sclera and vortex vein hypoplasia, and the secondary block of transscleral fluid outflow.

In types 1 and 2 UES, OCT/OCTA revealed marked thickening of the choroid and sclera (Figure 1), and at surgery, the sclera was abnormally rigid and unusually thickened to more than 2.5 mm. Histopathological studies showed disorganization of collagen fiber bundles and variation in size of collagen fibrils. These findings were consistent with previous studies (7–13, 17). Because of the histopathological abnormalities in the rigid and thickened sclera, fluid drainage was also compromised from the decreased function of uveoscleral outflow pathways, leading to excessive protein and fluid accumulation in the suprachoroidal space.

Previous studies have demonstrated excessive glycosaminoglycans accumulation in the matrix of the sclera, combined with defective vortex veins, resulting in decreased drainage of extravasated protein through channels of the transscleral outflow pathway (5, 24–26). However, in the present study, the histochemical examination showed the Alcian blue staining (glycosaminoglycans) in the matrix was negative in all eyes. These findings were not consistent with previous studies. Therefore, we believe that the theory of glycosaminoglycan deposition in the sclera of patients with UES needs further research and interpretation with caution.

Along with the main proposed mechanism which results in subretinal fluid accumulation, a second mechanism is the choroidal vein congestion caused by vortex vein compression by the thickened sclera (4). ICGA in types 1 and 2 demonstrated markedly diffuse choroidal hyperfluorescence during the early stage, and this finding persisted and increased in the late stage (Figures 3, 6). OCT/OCTA showed pachychoroid in type 1 and type 2, and the choroidal thickness was significantly increased. These findings suggest that there is marked hyperpermeability in the choroidal vessels and massive accumulation of fluid in the choroidal stroma. However, the choroidal thickness decreased with time during follow-up post-operatively, indicating that the speculation on the pathological mechanism of the disease is reasonable and indicating the effectiveness of this surgical treatment.

Ciliochoroidal detachment is manifested by the accumulation of fluid in the choroid, particularly in the suprachoroid, observed at the periphery for almost 360 degrees, even before the fundus changes detectably. During this period, the eye is asymptomatic and in the subclinical stage of UES. The condition usually occurs in the fellow eye in type 1, and is proven by OCT/OCTA, UBM, and ICGA. Following the development of the disease, long-term accumulation of the choroidal fluid decompensates the RPE and prevents inward to outward trans-RPE fluid flow through the pump mechanism of RPE (27). Subsequently, subretinal fluid accumulated and exudative retinal detachment occurred. In the clinical stage of UES, the ocular manifestations became apparent and symptomatic in eyes with both unilateral and bilateral involvement. These processes suggest that scleral abnormality is a major cause of the UES (Figure 8), and our study confirmed initial hypothesis (5) by Gass again.

Given the development of the disorder, surgical management of the sclera is reasonable (2, 3, 5, 6). In 1975, Shaffe (28) proposed that the cause of ciliochoroidal effusion associated with nanophthalmos is choroidal engorgement caused by impaired drainage of vortex veins because of the thick sclera characteristic of this disease. Brockhurst (3) subsequently reported the successful use of vortex vein decompression as a surgical remedy of nanophthalmic uveal effusions. However, the surgery was challenging to perform because considerable bleeding occurred during the removal of the sclera around the vortex vein. In 1983, Gass (5) suggested partial-thickness sclerectomies without vortex vein decompression in UES with nanophthalmos and reported successful effectiveness. Uyama (29) subsequently performed subscleral sclerectomy (sclerectomy under the scleral flap) without vortex vein decompression. This procedure preserved the scleral flap and the procedure was relatively complicated, and this may be related to recurrence post-operatively. In recent years, in Mansour's (29) small series of type 1 UES, faster resolution of subretinal fluid was reported with extensive circumferential scleral resection (90% thickness) without unroofing the choroid, allowing the removal of the scleral barrier to diffusion. They described the effectiveness with speed of resolution [within an average (±SD) of 13.9 (±8.7) days)]; however, we still believe with this procedure, there may be a risk of developing scleral staphyloma after resection because of the large-scale scleral removal. Therefore, we attempted to modify the Gass's techniques and performed minimum volume quadrantic lamellar-sclerectomy with sclerostomy without decompression of the vortex vein, retaining the stable IOP and avoiding hemorrhage during the surgery. The rationale for abovementioned procedures were similar to that of trabeculectomy to create a bypass outflow route for the aqueous humor.

The nature of the scleral abnormality in IUES has not been clarified. There is evidence that vortex vein obstruction also plays a role in the pathogenesis of UES in normal-sized eyes (7). In our series, signs of increased uveal venous pressure, such as dilated episcleral veins, were obviously present in most type 2 patients, which may be associated with the sclera and increased resistance to transscleral outflow of the intraocular fluid. Uyama (30) found blood in Schlemm's canal, and on intraoperative examination, the vortex veins were thought to be reduced in number and/or caliber in 68% of eyes. Furthermore, signs of uveal congestion observed in type 2 patients in our study decreased or disappeared after scleral surgery. This implies that vortex vein obstruction is at least partially a secondary change, resulting possibly from scleral swelling induced by high protein concentration (5). In addition, in our series, type 2 patients had bilateral disease, which may imply the associated systemic factors.

Based on the previous reports indicating that the proteoglycan composition of the surrounding matrix controlled the size and organization of collagen fibers (31, 32), several reports speculate that a defect in mucopolysaccharide metabolism may be the cause of abnormalities such as thickened sclera or permeability in this disorder (7, 9, 33, 34). Of interest, in this regard, was the occurrence and successful treatment of ciliochoroidal effusion by combined sclerectomies and sclerostomies in a patient with systemic mucopolysaccharidosis type II (Hunter's syndrome), a disorder in which the sclera is thickened by the deposition of mucopolysaccharide (8). This type previously was categorized as IUES and was treated in the same manner as nanophthalmos. Interestingly, in our series, the type 2 UES accompanied different systemic symptoms, which may be categorized as the systemic disease-associated factors to UES; thus, “idiopathic/primary UES” in the true sense is still controversial.

Thus, in IUES, transscleral protein transport impairment seems to be the primary pathophysiologic factor and uveal congestion plays a secondary role. Based on the successful use of Gass's technique in patients with nanophthalmic uveal effusion (19, 20, 35), Allen (20) and Johnson (21) suggested that the same pathophysiology and surgery may be applied in IUES.

ICGA revealed that a subclinical condition in the choroid lasts for a long period after surgery. Recurrence of retinal and ciliochoroidal detachment sometimes occurs. When the effects of draining choroidal fluid cease, reoperation is necessary and can be performed easily without complications. In our small number of patients with recurrence, reoperation was favorable. Thereafter, the suprachoroidal fluid spontaneously drained and retinal detachment gradually resolved.

In this study, we achieved a successful outcome with this procedure without any complication in all eyes with types 1 or 2 UES. This procedure was easy to perform, and the outcomes were excellent. For type 1 nanophthalmos, secondary angle closure cause by uveal effusion occurs because of fluid accumulation in the supraciliary space extending anteriorly from the suprachoroidal space (36). UBM demonstrated that the ciliary body is anatomically hinged to the eye wall at the scleral spur, and accumulation of supraciliary fluid leads to detachment and anterior rotation of the ciliary body. The mass effect causes forward displacement of the lens–iris diaphragm, leading to a shallow anterior chamber, aqueous misdirection, appositional angle closure, and elevated IOP. In our experience, it is necessary to perform YAG laser iridectomy before scleral surgery to reduce IOP and avoid intraoperative complications, such as suprachoroidal hemorrhage.

In conclusion, our results revealed that abnormal sclera and increased resistance to transscleral outflow of intraocular fluid is the primary cause of types 1 and 2 UES, and creation of a bypass outflow route for intraocular fluid by lamellar-sclerectomy with sclerostomy is the rationale for surgical treatment of this condition. Minimum volume quadrantic lamellar-sclerectomy with sclerostomy is highly recommended as the first-choice treatment and is effective. Although we found no significant correlation between the lasting term of pre-operative RD and final BCVA, the excellent results with the modified surgical technique performed in our cases and the visual recovery seen in many patients indicates that surgical intervention as soon as macular function is threatened by subretinal fluid accumulation is the key to successful treatment. Further systemic and genetic studies associated with scleral abnormality should be helpful in understanding the precise cause of primary UES.

## Data Availability Statement

The raw data supporting the conclusions of this article will be made available by the authors, without undue reservation.

## Ethics Statement

The studies involving human participants were reviewed and approved by Medical Ethics Committee of the Beijing Tongren Hospital. The patients/participants provided their written informed consent to participate in this study. Written informed consent was obtained from the individual(s) for the publication of any potentially identifiable images or data included in this article.

## Author Contributions

WW and LY: examination of the patient and interpretation of results. NZ and XX: interpretation of results, writing, and reviewing of the manuscript. All authors read and approved the final manuscript.

## Funding

This study was funded by the National Natural Science Foundation of China (No. 81272981) and the Beijing Natural Science Foundation (No. 7151003) provided financial support.

## Conflict of Interest

The authors declare that the research was conducted in the absence of any commercial or financial relationships that could be construed as a potential conflict of interest.

## Publisher's Note

All claims expressed in this article are solely those of the authors and do not necessarily represent those of their affiliated organizations, or those of the publisher, the editors and the reviewers. Any product that may be evaluated in this article, or claim that may be made by its manufacturer, is not guaranteed or endorsed by the publisher.

## Supplementary Material

Supplementary Figures 1, 2 UES in type 1. The restoration of photoreceptor and RPE damage, flattening of crowded macular morphology, and expansion of attenuation of FAZ were observed by SS-OCT/OCTA with time during follow-up post-operatively.
