# Supplementary figures and images for: Uveal Effusion Syndrome: Clinical Characteristics, Outcome of Surgical Treatment, and Histopathological Examination of the Sclera
Source: Front Med (Lausanne). 2022 Jun 9;9:785444. doi: 10.3389/fmed.2022.785444 (PMC9218343; doi:10.3389/fmed.2022.785444)

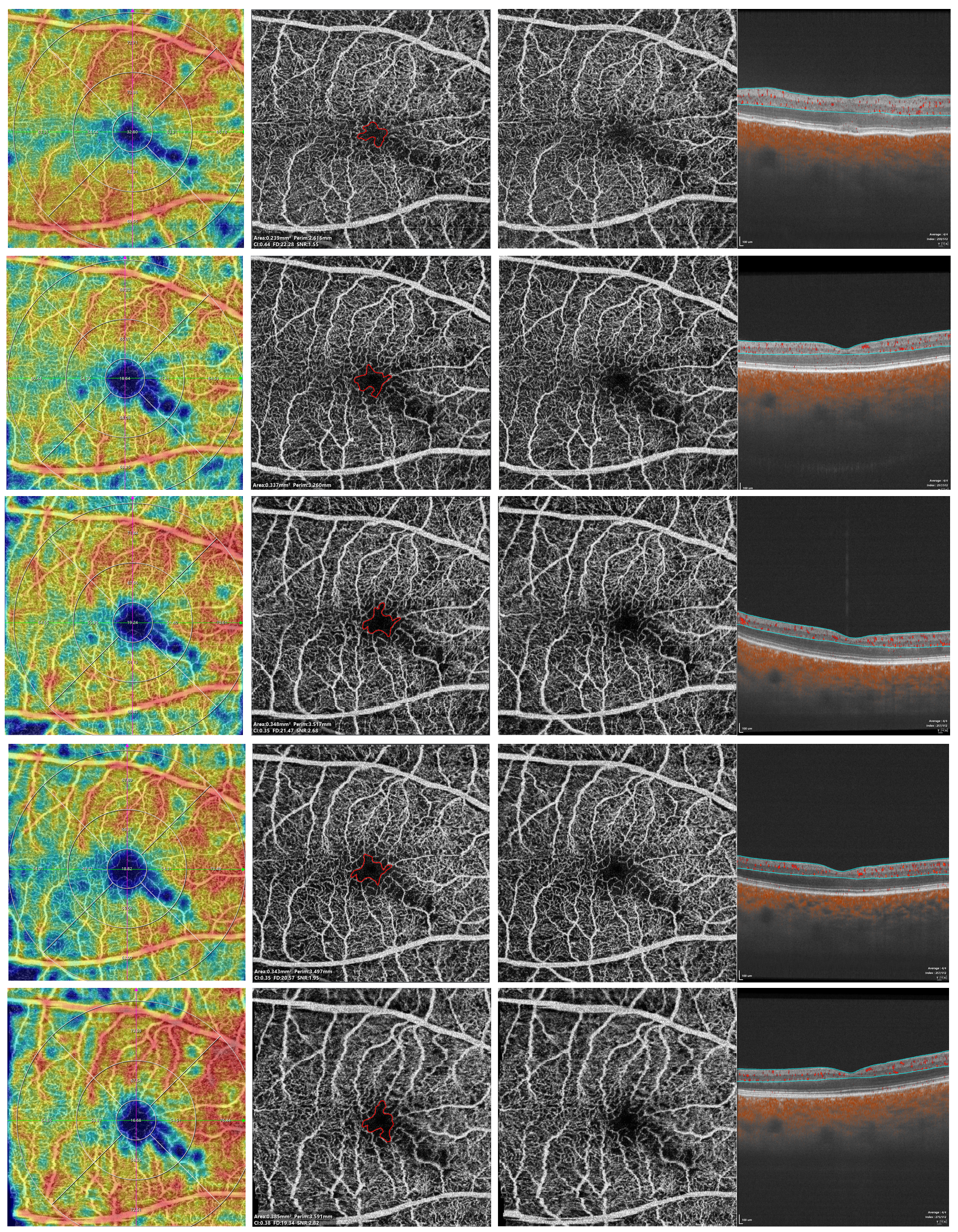

Supplement: Supplementary file 1 [file Image_2.tif]

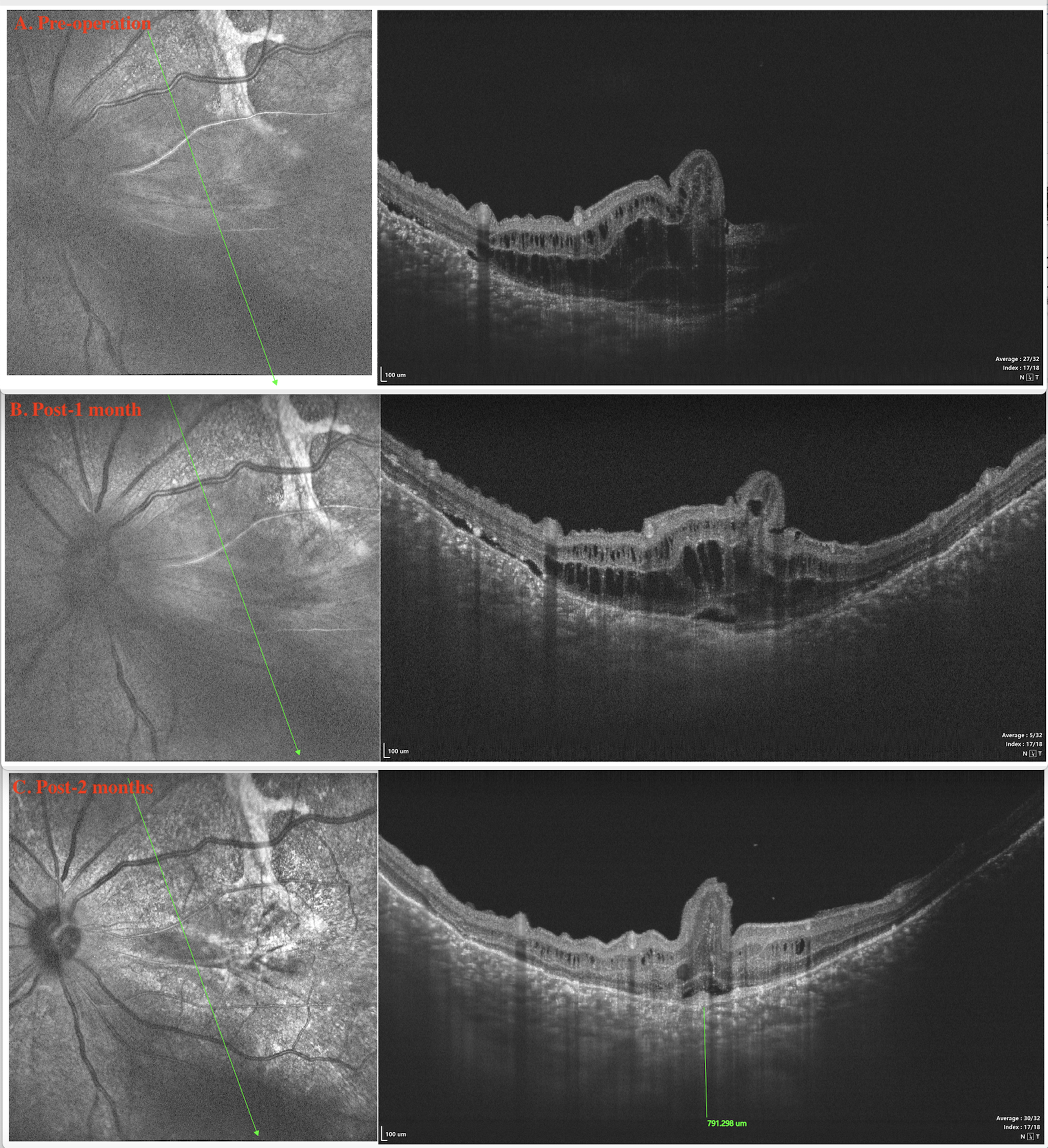

Supplement: Supplementary Figures 1, 2 — UES in type 1. The restoration of photoreceptor and RPE damage, flattening of crowded macular morphology, and expansion of attenuation of FAZ were observed by SS-OCT/OCTA with time during follow-up post-operatively. [file Image_1.PNG]
